# Vesicoovarian Fistula on an Endometriosis Abscessed Cyst

**DOI:** 10.1155/2014/240596

**Published:** 2014-07-24

**Authors:** C. Tran, M. Even, M. Carbonnel, F. Preaux, F. Isnard, A. Rault, M. Rouanne, J. M. Ayoubi

**Affiliations:** Department of Gynecology Obstetrics, Foch Hospital, 40 rue Worth, Suresnes, France

## Abstract

We report the case of a patient who developed a vesicoovarian fistula on an endometriosis abscessed cyst. The patient presented with an advanced endometriosis stage IV complicated with a right ovarian abscessed cyst of 10 cm. A first coelioscopy with cystectomy was realized. After surgery, a voiding cystography highlighted a fistula between the ovarian abscess and the bladder. A second surgery by median laparotomy was realized with the resection of the right ovarian abscess and the resection of vesical fistula.

## 1. Introduction

Numerous complications of the endometriosis exist. The vesicoovarian fistula is a very rare case; only two cases have been documented. We are going to present the case of a patient who developed a vesicoovarian fistula on an endometriosis abscessed cyst.

## 2. Case Study

A 31-year-old patient was treated by the gynecology obstetrics department for a 10 cm right ovarian endometriosis cyst before going into* in vitro* fertilization.

She presented with in her medical history a uterine malformation with a complete compartmentalized uterus and a complete vaginal partition, an ankylosis spondylitis (AS), and endometriosis. She had two laparoscopies in 2007 as part of her infertility check-up. The first one in June showed a severe endometriosis stage IV with ice-cold pelvis and a lot of adhesions. Nothing was done during this surgery, and a second surgery was subsequently performed in December after six months of treatment by LHRH analogs.

This second surgery resulted in adhesiolysis and an incomplete resection of bilateral endometriosis cysts, confirmed by histopathology.

The patient was in the medical assisted procreation (MAP) program. She was expecting at the end of the second attempt of* in vitro* fertilization (IVF). She had an urgent C-section in 2009 at 36GW + 3 days due to a premature rupture of membranes with beginning of a maternofoetal infection. In February 2012, she had a second trial of IVF treatment hoping for a second child.

During the fertilization check-up, a 10 cm right ovarian endometriosis cyst was discovered. The patient was sent for cystectomy before IVF. She had an asymptomatic recurrent urinary infection and turbid urines for several months. She did not have abdominal pain or fever. She denies dysmenorrhea or dyspareunia. Elevated C-reactive protein (CRP) was attributed to her periodic inflammatory reactions of AS.

**Figure 1:**
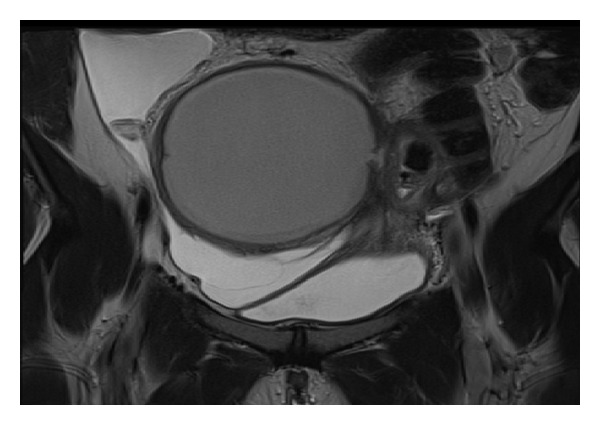
RMI coronal view, T2 sequence, and abscess with fistula on its left side.

The preoperative magnetic resonance imaging (MRI) showed a 11 × 9 cms endometriosis cyst of the right ovary, with a doubtful fistula in the bladder on its left side (Figure 1).

A right hydrosalpinx of 6 mm was also identified below the voluminous cyst. Numerous adhesions and a deep endometriosis invading the two-uterosacral ligaments had been found. A cystoscopy was performed before surgery but no fistula was found.

The patient was treated with LHRH analogs and a coelioscopy was scheduled in January 2013. The patient had no fever. Her blood test was normal with 7880 white blood cells but the culture of the CBEU turned out to be* E. coli*.

Upon catheterization, the patient still had cloudy urines. During the laparoscopy, numerous adhesions were identified, preventing a complete exploration. During the right ovary cystectomy, there was a leakage of fluid.

The cyst was partially resected and the abscess was drained.

Samples were sent to bacteriology. Her peritoneal fluid had* Escherichia coli* which was sensitive to ofloxacin. An appendectomy was performed at the same time. No fistula in the bladder was visualized. The patient was initially put under quadriantibiotic treatment (ofloxacin, metronidazole, ceftriaxone, and gentamicin). The postoperative blood test found 9300 white blood cells of which 83% were neutrophils and her CRP was 114. The patient was discharged after three days with a protocol of antibiotics. Twenty-two days after being released, she came back for her follow-up. Her follow-up CRP, 22 days after discharge, was 380 and she still had cloudy urines. No symptoms of fever were found.

**Figure 2:**
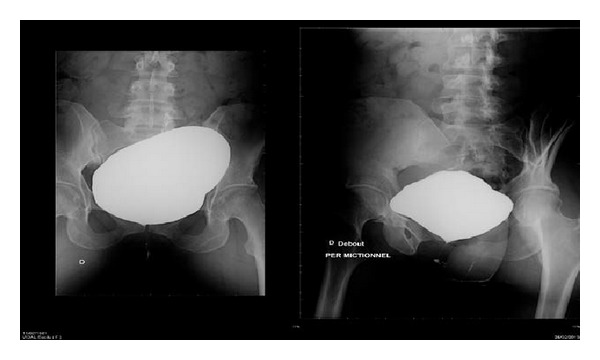
Retrograde cystography showing the vesical fistula.

A retrograde cystography highlighted a fistula between the abscess, which had been reconstituted (measuring 8.5 cm), and the bladder (Figure 2).

The control CBEU did not find bacteria in the direct examination or in the culture. A new intervention by median laparotomy was scheduled in April 2013. At this time, she did not undergo hormonal treatment before the surgery.

Upon exploration numerous adhesions were found between the sigmoid colon and the cyst. It completely covers the intestinal loops, the small intestine, and cecum. There were also severe adhesions from sigmoid area up to the posterior pelvic wall due to severe persistent endometriosis; Douglas' pouch appears shielded by these adhesions.

After a difficult adhesiolysis, the uterus, the sigmoid, the right ovary, and the bladder were identified and visualized. An injection of a blue dye confirmed the presence of a very small caliber fistula about 1-2 mm, located at the top left quadrant of the bladder. It is a fistula of very small caliber to about 1-2 mm oblique pinhead. The intervention allowed the resection of the right ovarian abscess and the debridement of the vesical fistula. A mobilization of the bladder was done after the opening of the retropubic space. Finally, a withdrawal of a 1 cm bladder patch and suture of the bladder in 2 plans were performed. Suction and drainage had been implemented.

Histopathological findings were performed on the right ovarian endometriosis cyst and the bladder fistula.

The surgery was uneventful with no complications. The patient was discharged after removal of the Foley catheter, eight days after surgery. The uroscan was normal. Her urine evidently became clear. No abscess or infection was noted.

## 3. Comment

The vesicoovarian fistula is an exceptional case. Only two cases have been reported. The first case was a fistula caused by an ovarian abscess linked to salpingitis in 1990 [1].

The second case was described in Japan in 1997 [2] wherein a fistula resulting from an adnexal abscess caused by endometriosis has reached the ovaries. The case was about a 44-year-old patient with IUD, admitted for feverassociated with urine infection.

Acystoscopy revealed a mass at the posterior wall of the bladder and the MRI showed a fistula between this mass and the bladder.The anatomopathology reported that the fistula was caused by endometriosis.

The case that we reported is an atypical case due to its poor symptomatology. Indeed, the patient had no fever and did not present any pelvic pain.

There was only an isolated increase of CRP which had been attributed to inflammatory response of AS.

Furthermore, the diagnosis of fistula was difficult to make. The preoperative cystoscopy did not demonstrate the presence of the fistula, neither did the laparoscopy. The MRI was suspicious for the presence of a fistula but the diagnosis was confirmed by a voiding cystography. Fistulas are difficult to diagnose, especially when they are very small. The voiding cystography remains the gold standard of diagnosis. This procedure gives pressure to the bladder and highlights fistulas of very small caliber.

The cause of the fistula is uncertain in our case. Was the fistula due to ovarian abscess draining of the surrounding organs, in this case, the bladder? Or was the fistula due to endometriosis complications by an abscess as a consequence of the urine contamination?

Furthermore, the origin of the abscess remains unclear: was it connected to the draining of the cyst for the IVF two years previously? Or did it result from a spontaneous secondary infection of the endometriosis cyst, without any iatrogenic intervention associated?

The vesical fistulas in the bladder in female patients are mostly iatrogenic, as a result of a medical history of pelvic surgery, in particular C-sections for vesicouterine fistulas or radiotherapy [3]. Infections or inflammatory diseases such as the endometriosis can also be responsible for the development of fistulas without initial surgical intervention [4]. However the spontaneous fistulas are very rare in the endometriosis. They are mostly iatrogenic, due to late surgical complications, linked to perforations during surgery.

Endometriosis fistulas are hormone dependent. It was reported that some rare cases of vesicouterine fistulas close after a medical treatment by LHRH analogs [4]. Indeed, LHRH analogs can lead to a secondary amenorrhea and the atrophy of the endometrial epithelium results in the closure of the fistula. Furthermore, the end of the menstrual blood flow through the fistula could facilitate its spontaneous closure [5]. However, these studies relate only to the vesicouterine fistulas and the number of cases is insufficient to make a conclusion about the efficiency of those treatments. Concerning our case, LHRH analogs had been established six months before the first intervention. Thus, the medical treatment did not allow by itself the regression of the fistula [6].

Surgical treatment with the abscess resection and the repair of the fistula was the only treatment that allowed complete recovery for the patient. Surgery remains the treatment of choice for endometriosis fistulas. If the laparoscopy did not seem appropriate in our case because of the very small size of the fistula, this surgical technique is recommended as a first intervention for the treatment of endometriosis fistulas to avoid the development and the extension of the fibrosis [7]. However, some teams recommend waiting for six weeks before surgery because some of the smallest fistulas can close spontaneously.

Some risk factors of recurrence of fistulas after surgical treatment had been identified: advanced endometriosis (stage III or IV), advanced age patients, presence of many pelvic adhesions, larger endometriomas or deep nodules, a failure of previous medical treatment, or an incomplete surgical resection [8–10].

We reported a very rare case of endometriosis complications with a vesicoovarian fistula. Although fistulas are rare, it is necessary to remain attentive to symptoms that make the diagnosis possible, to be able to ensure adequate treatment. To avoid recurrences and complications, a surgical complete excision is recommended in these cases.
